# Comparative effectiveness of smartphone healthcare applications for improving quality of life in lung cancer patients: study protocol

**DOI:** 10.1186/s12890-022-01970-8

**Published:** 2022-05-02

**Authors:** Jang Ho Lee, Jae Hwa Jeong, Wonjun Ji, Hui Jeong Lee, Yura Lee, Min-Woo Jo, Seockhoon Chung, Sung-Cheol Yun, Chang-Min Choi, Geun Dong Lee, Sei Won Lee, Jong Won Lee

**Affiliations:** 1 Division of Pulmonology and Critical Care Medicine, Department of Internal Medicine, Asan Medical Center, University of Ulsan College of Medicine, 88 Olympic-ro 43-gil, Songpa-gu, Seoul, 05505 Republic of Korea; 2 Department of Thoracic and Cardiovascular Surgery, Asan Medical Center, Ulsan University College of Medicine, 88 Olympic-ro 43-gil, Songpa-gu, Seoul, 05505 Republic of Korea; 3 Division of Breast Surgery, Department of Surgery, Asan Medical Center, University of Ulsan College of Medicine, Songpa-gu, Seoul, Republic of Korea; 4 Department of Preventive Medicine, University of Ulsan College of Medicine, Songpa-gu, Seoul, Republic of Korea; 5 Department of Psychiatry, Asan Medical Center, University of Ulsan College of Medicine, Seoul, Republic of Korea; 6 Department of Clinical Epidemiology and Biostatistics, Asan Medical Center, University of Ulsan College of Medicine, Seoul, Republic of Korea; 7 Department of Oncology, Asan Medical Center, University of Ulsan College of Medicine, Seoul, Republic of Korea

**Keywords:** Lung cancer, Quality of life, Exercise, Smartphone application, Pulmonary rehabilitation

## Abstract

**Background:**

Although pulmonary rehabilitation is helpful for patients following lung cancer surgery, rehabilitation is not widely available, due in part to a lack of medical resources. Recent developments in digital health care have overcome the space limitations associated with in-person health care. This study will evaluate and compare the efficacy of three different smartphone healthcare systems in patients with lung cancer.

**Methods:**

This single center randomized controlled study is designed to evaluate the efficacy of digital healthcare applications for lung cancer patients after thoracoscopic lung resection. A total of 320 patients will be enrolled and randomized 1:1:1:1 into four different groups, with one group each using the smartphone applications NOOM, Walkon, and Efilcare and the fourth being the control group without intervention. Questionnaires will be administered to patients at baseline and after 3, 6, and 12 months. The primary endpoint will be the score on the EuroQol five-dimension index. Secondary endpoints will include other questionnaires about quality of life and dyspnea.

**Discussion:**

This prospective randomized controlled study may allow assessments and comparisons of the efficacy of various smartphone applications in patients who undergo lung cancer surgery. This process may enable the introduction of healthcare interventions that maintain quality of life in patients with lung cancer.

*Trial registration* CRIS, KCT0005447. Registered 06 October 2020, https://cris.nih.go.kr/cris/search/detailSearch.do/19346

## Background

Lung cancer is one of the most commonly diagnosed malignancies and the leading cause of cancer-associated deaths worldwide [1]. The overall survival of patients with lung cancer has been significantly improved by the use of surgery, neoadjuvant and/or adjuvant chemotherapy, and radiotherapy, with survival being especially improved in patients with early stage non-small cell lung cancer [1]. Although survival is the most important goal, maintaining of quality of life and exercise capacity are also important. Increased exercise capacity and better quality of life have been associated with favorable long-term outcomes in various diseases [2–4]. Surgical resection of lung tumors reduces lung volume, resulting in reduced exercise capacity and poorer quality of life. Perioperative management of lung cancer patients should therefore focus on quality of life [5–7]. Pulmonary rehabilitation of patients who have undergone lung resection was found to improve their exercise capacity, pulmonary function, and quality of life [8, 9]. Pulmonary rehabilitation, however, is limited by a scarcity of medical resources and difficulties supervising adequate pulmonary rehabilitation [10].

Proper diet and exercise after anti-cancer treatment are important for lung cancer patients [11–13]. Although the ability of clinicians to supervise pulmonary rehabilitation and provide education about diet is limited by lack of time and interest, digital interventions are regarded as effective alternatives to in-person management [14–17]. Digital interventions through smartphone applications are preferred owing to easy accessibility and expandability, including the ability of these applications to provide a direct coaching system and important information [18]. Although most studies assessing the efficacy of smartphone applications have been performed in patients with chronic diseases, these applications may also help patients with lung cancer who plan to undergo surgery and require postoperative lifestyle management.

The present study is designed to evaluate the efficacy of smartphone healthcare applications for lung cancer patients following thoracoscopic lung resection. Three smartphone applications and a control group will be compared using the results of pulmonary function tests and exercise capacity, as well as questionnaires related to patient quality of life and psychological distress.

## Methods/design

### Study design

This study is designed as a single center randomized controlled study evaluating the efficacy of digital healthcare applications for lung cancer patients who undergo thoracoscopic lobectomy. Patients will be recruited at Asan Medical Center, a university affiliated 2700-bed tertiary medical center. A total of 320 patients will be enrolled. The study duration is 1 year, and all enrolled patients will be evaluated at outpatient clinics by attending clinicians at baseline and at 3, 6, and 12 months after surgery. The study protocol has been approved by the Institutional Review Board of Asan Medical Center (IRB no. 2020-1015), and written informed consent will be obtained from all participants by attending investigators and research assistants.

### Study participants

Patients scheduled to undergo a planned thoracoscopic lobectomy for lung cancer will be screened by attending clinicians. Patients will be included if they (1) are aged 20–70 years; (2) have been pathologically diagnosed with primary lung cancer; (3) are scheduled for lobectomy with a thoracoscope; (4) have no evidence of lymphatic or distant metastasis; and (5) provide written informed consent. Patients will be excluded if they (1) have any evidence of metastasis; (2) have a history of segmentectomy or wedge resection for lung cancer; (3) are changed to open thoracotomy during surgery; (4) are scheduled for neoadjuvant or adjuvant chemotherapy or radiotherapy around the time of surgery; (5) are pregnant or breast-feeding; (6) have a chronic respiratory disease, including asthma, pulmonary tuberculosis or chronic obstructive pulmonary disease; (7) have a history of diagnosis of other malignancies within 5 years; and (8) have a disability that would make exercise difficult. Adequate patients will be enrolled in this study based on the attending physicians’ and corresponding author’s judgements.

### Randomization and intervention

**Fig. 1:**
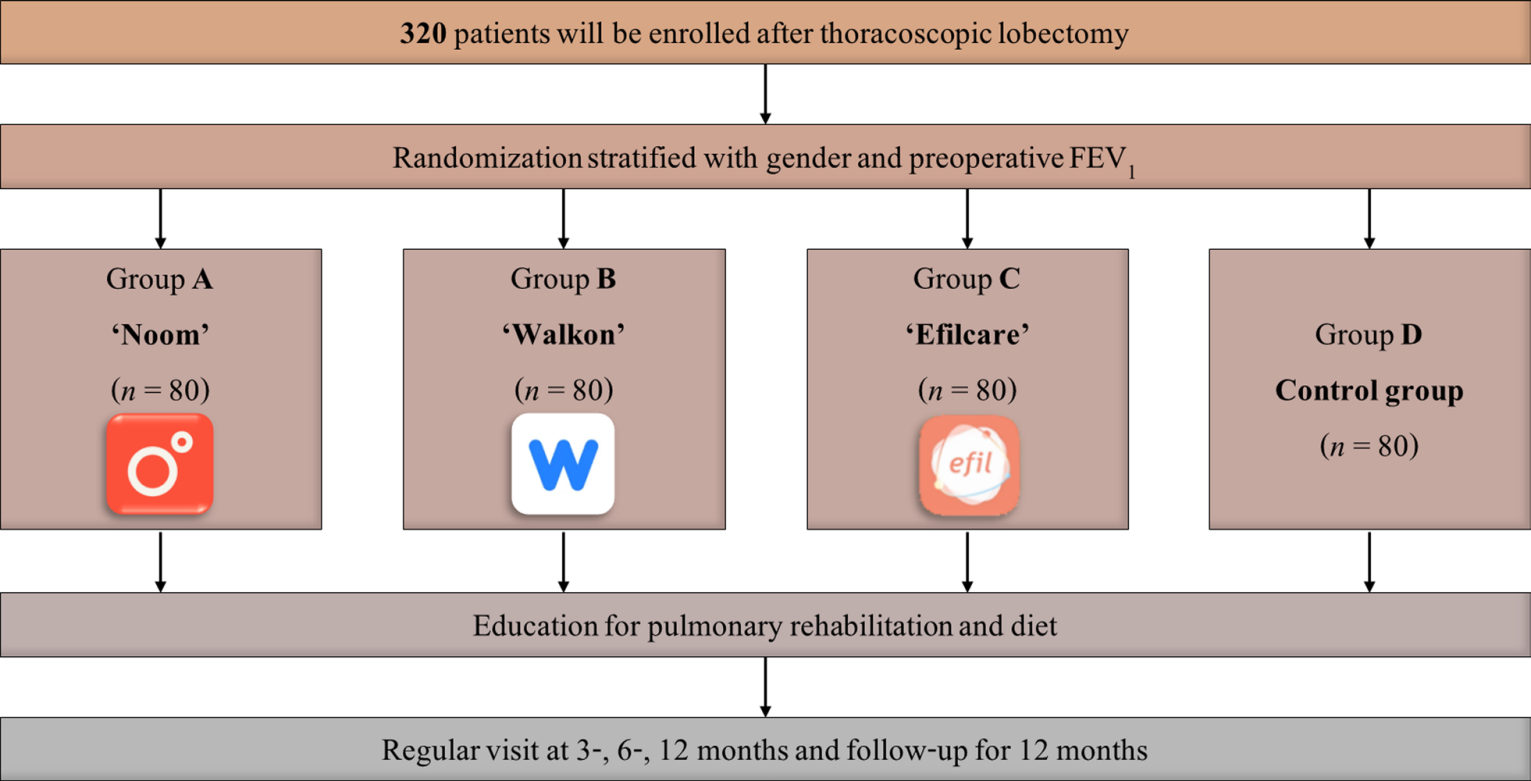
Study flow

The study flow is presented in Fig. 1. The enrolled patients will be stratified according to gender and forced expiratory volume in 1 s (≥ 60% or < 60% of the predicted normal value). The patients will be subsequently randomized 1:1:1:1 to four groups. Allocation to the control or intervention group will be based on the pre computer-generated random number list by a statistician. Following thoracoscopic lobectomy for lung cancer and stabilization, all enrolled patients will participate in education sessions for pulmonary rehabilitation. During these sessions, specialists will teach methods of stretching, aerobic exercise, and muscle training, and recommend an adequate diet [10]. In group A, patients will use the smartphone application NOOM, for 12 weeks. After that, the patients will try to maintain the modified lifestyle. Patients will use Walkon in group B and Efilcare in group C during study periods. Patients in group D will be the control group without any intervention. Enrolled patients will undergo the measurement and questionnaires at index date and after 3, 6, and 12 months. During each visit, attending clinicians will motivate and educate the patients. Allocation concealment will be guaranteed by opaque, sealed envelopes.

### Digital intervention with smartphone applications

**Fig. 2:**
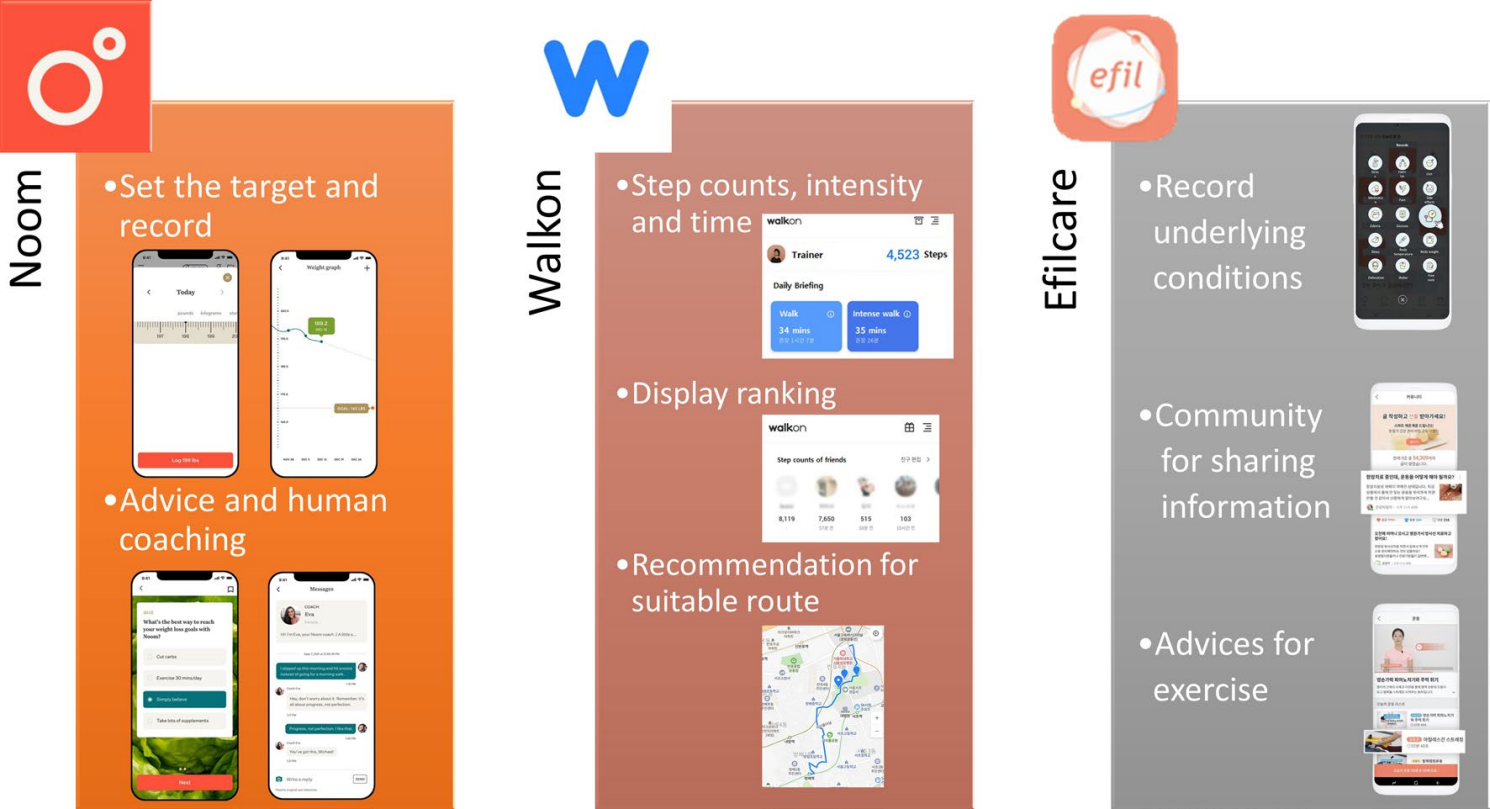
Description of the three smartphone applications tested in this study. (left) NOOM, (center) Walkon, (right) Efilcare

The NOOM, Walkon, and Efilcare smartphone applications are illustrated in Fig. 2. NOOM is a worldwide application in which users initially record their gender, height, weight, and age and set a target body weight. The application introduces adequate methods of diet and exercise. Users continuously record their body weight and patterns of diet and exercise. Based on these records, human coaches regularly contact the users for supervision and advice via messages.

The Walkon application measures step counts, and the intensity of walking exercise based on time and velocity. It also encourages users by displaying ranking based on walking record, recommending suitable routes for walking, and providing various benefits as compensation.

Users of the Efilcare application record their underlying disease, degree of stress, diet and exercise patterns, body weight, body temperature, blood glucose, and blood pressure. Based on these parameters, the application recommends proper methods of exercise and provides pre-written information, including about lung cancer.

### Data collection and study outcome

At screening, researchers will record patients’ baseline characteristics, including age, sex, smoking history, European Cooperative Oncology Group Performance Status, underlying disease, and previous cancer history. In addition, lung cancer-related information, including pathologic diagnosis, clinical and pathologic stage, and surgery record, will be recorded.

The primary study endpoint will be score on the EuroQol five-dimension (EQ-5D) index, which measures patient quality of life [19]. Secondary endpoints will include body weight and body mass index, as well as measures of pulmonary function and exercise capacity, such as pre- and post-bronchodilator pulmonary function tests, diffusion capacity and 6-min walk tests. Other secondary endpoints will include quality of life questionnaires to evaluate quality of life (Distress Thermometer, Health-Related Quality of Life Instrument with 8 Items, Patient Health Questionnaire-9, Fear of Progression Questionnaire-Short Form, Cancer-related Dysfunctional Beliefs about Sleep, Insomnia Severity Index) and dyspnea degree (modified Medical Research Council dyspnea scale). The timeline of data collection is presented in Table 1.

**Table 1:** Timeline of data collection

	Screening and enrollment Index date	Visit 1 3 months	Visit 2 6 months	Visit 3 12 months
Medical history	●			
Chest CT and laboratory tests	●	●	●	●
Informed consent	●			
Body measurements	●	●	●	●
PFT and 6MWT	●	●	●	●
Questionnaires	●	●	●	●

*6MWT* 6-min walk test, *CT* computed tomography, *PFT* pulmonary function test

### Data management and monitoring

Discriminable data will be removed, replaced with the anonymised study identifier, and transferred to the principal investigator. Questionnaire data will be collected and sent to statisticians for further analysis. Data collection will be performed by trained research assistants. Access to the data will be limited to the authors and research assistants. Authors will perform data management under the responsibility of corresponding author.

During the study period, the process of each task will be periodically monitored and evaluated according to the timeline. An internet meeting consisting of the corresponding author, attending investigators, research assistants will be held to monitor the process. In this meeting, all researchers will discuss about the protocol modification, if needed.

### Sample size calculation

We assumed for sample size calculation that effective postoperative rehabilitation could restore the quality of life to a similar level before surgery. A previous lung cancer study showed that the preoperative and postoperative EQ-5D indices were 0.81 ± 0.19 and 0.74 ± 0.11, respectively [20]. Additionally, a minimal clinically important difference in EQ-5D has been observed between 0.03 and 0.05 [21]. Because patients were classified into four groups, the margin of type 1 error was set at 0.008. Calculations showed that a minimum of 76 patients in each group was required to detect a significant difference at the 0.008 level with a power of 0.9 and a dropout rate of 25%.

### Statistical analysis

SAS (SAS Institute Inc., Cary, NC) and R (version 4.1.2; R Development Core Team, Vienna, Austria) software will be used for all statistical analyses. Continuous variables will be presented as means ± standard deviations or median [interquartile range] and compared by Student’s *t*-test or the Mann–Whitney U-test. Categorical variables will be presented as numbers and percentages and compared by the χ^2^ test or Fisher’s exact test. All tests of significance will be two-sided, with *P* < 0.05 defined as statistically significant.

## Discussion

Improvements in exercise capacity and quality of life have been associated with more favorable outcomes in patients with lung cancer [22–24]. Recent advances in digital health care have included smartphone application based pulmonary rehabilitation, which is effective in improving exercise capacity and symptoms in patients with advanced lung cancer [25]. This study is designed to expand the therapeutic potential of smartphone application based pulmonary rehabilitation to patients with early-stage lung cancer who are scheduled to undergo thoracoscopic lung resection and will have reduced lung function and volume after surgery. Although this type of clinical trial is currently uncommon, these trials will likely be more common in the near future, as these interventions can overcome the limitations of medical care within a hospital. This study is designed not only to evaluate the efficacy of smartphone application, but also to compare the efficacy of three different  types of smartphone applications. Patients with early-stage lung cancer usually undergo surgery with curative intent, making management that facilitates prompt recovery more important.

Due to the scarcity of medical resources, most clinicians are interested in acute phase treatment. However, sustainable health care management, such as lifestyle modifications outside the hospital, is also important. Digital intervention can be effective in optimizing patient management. Smartphone applications have several advantages, including their relatively high adherence rates compared with other system. The rates of adherence for weight loss was 93% for smartphone applications, compared with 55% for websites and 53% for diaries [26]. In addition, subjects can monitor their step counts and intensity of walking just by carrying a smartphone [27]. Smartphone applications also allow feedback on a patient's lifestyle, thereby motivating patients [18]. Other advantages of digital interventions using smartphone applications include relatively low medical costs and newly developed expandability [18].

Diet and exercise are also essential for patients after anti-cancer treatment [11]. For these items with smartphone application were well studied in obesity. In these studies, smartphone applications are effective in treating obesity, by reducing and maintaining target body weight [28–30]. The effectiveness of smartphones in obesity resulted from daily life tracking and active feedback on subjects’ diet and exercise patterns. Before discharge, many hospitals instruct patients on diet and exercise after surgery. However, patient compliance with these instructions can be evaluated only during hospital visits, making continuous education and motivation with appropriate feedback difficult. A finding, that the use of smartphone applications can modify the lifestyle of patients with lung cancer, would support the future use of these applications, similar to those in obesity.

The present study will examine three different smartphone applications for lifestyle modification. These applications will likely have effects on patients in different ways. Those randomized to NOOM will be instructed to monitor their diet and exercise patterns every week and encouraged by text messages from human coaches to achieve their target body weights. This protocol assumes that contact via text will produce effects similar to face-to-face contact with supervisors. Users of Walkon will be able to confirm daily step count and the time and intensity of walking. Walkon displays rankings within a group, suggesting that this application will motivate the users through competition. Users of Efilcare will receive pre-written information based on daily records, thus providing individualized information to users. Although each application has been studied in various diseases, few studies to date have compared their efficacy.

In conclusion, this randomized controlled study is designed to assess the efficacy of smartphone healthcare applications for patients who have undergone thoracoscopic lung resection. In addition, this study will investigate the differences in efficacy among the three applications. Because these applications act via different mechanisms, they may identify the optimal application for patients with lung cancer. This study will likely provide evidence about the efficacy of smartphone healthcare application for digital lifestyle management and improvements in quality of life and exercise capacity via publication. The results of this study may expand the use of digital healthcare application to patients with other types of cancer.

### Trial status

Recruitment has started and is ongoing since November 2020.

## Data Availability

The datasets used and analyzed during the current study will be available from the corresponding author on reasonable request.

## Abbreviation

EQ-5D index: EuroQol five-dimension index
